# Radiation recall gastritis secondary to combination of gemcitabine and erlotinib in pancreatic cancer and response to PPI - a case report

**DOI:** 10.1186/s12885-016-2616-3

**Published:** 2016-08-02

**Authors:** Seong Ji Choi, Hyo Jung Kim, Jae Seon Kim, Young-Tae Bak, Jun Suk Kim

**Affiliations:** 1 Department of Internal Medicine, Korea University Guro Hospital, Korea University College of Medicine, Seoul, South Korea; 2 Division of Gastroenterology, Department of Internal Medicine, Korea University Guro Hospital, Korea University College of Medicine, Seoul, South Korea; 3 Division of Oncology, Department of Internal Medicine, Korea University Guro Hospital, Korea University College of Medicine, Seoul, South Korea

**Keywords:** Radiation recall, Gastritis, Erlotinib, Pancreatic cancer, Radiotherapy

## Abstract

**Background:**

Radiation recall gastritis is rare but can be induced after concurrent chemoradiation for pancreatic cancer. We report a patient with pancreatic cancer who developed radiation-recall gastritis related to a combination of gemcitabine and erlotinib.

**Case presentation:**

A 54-year-old female with unresectable pancreatic cancer received gemcitabine in combination with radiation therapy followed by chemotherapy with gemcitabine and erlotinib. After completing 2 cycles of chemotherapy, the patient had epigastric pain, nausea, and vomiting. Abdominal computed tomography (CT) scan revealed diffuse wall thickening of the stomach, and esophagogastroduodenoscopy (EGD) showed multiple gastric ulcers. The patient was treated with proton pump inhibitors (PPI) and was continued on maintenance chemotherapy. Two months later, the patient presented with the similar symptoms and persistent gastric ulcers were observed during subsequent EGD. Nevertheless, the patient’s symptom had resolved with PPI therapy. Thus, the patient underwent maintenance chemotherapy with gemcitabine and erlotinib for additional 4 cycles. Eventually, follow-up abdominal CT Scan and EGD at 6 months demonstrated resolution of the gastric ulcers.

**Conclusions:**

Physicians should be aware of the possibility of radiation recall gastritis associated with a combination of gemcitabine and erlotinib. Administration of PPIs may mitigate the adverse effects of gemcitabine and erlotinib in the presence of radiation recall gastritis; however further studies are warranted.

## Background

Radiation recall is known as chemotherapy-triggered inflammatory reaction in previously exposed areas to irradiation but the mechanism is poorly understood [1]. Radiation recall gastritis can be occurred in pancreatic cancer [2]. Radiation recall gastritis due to combination of gemcitabine and erlotinib has not been reported in the literature. There are only two case reports, one is gemcitabine-related and the other is erlotinib-associated radiation recall gastritis [2, 3].

We experienced a case of pancreatic cancer with radiation recall gastritis after combination therapy of gemcitabine and erlotinib, but exhibited gradual improvement along with proper maintenance the combination chemotherapy and PPI (proton pump inhibitor).

## Case presentation

A 54-year-old woman with no medical history presented with a complaint of periumbilical pain and weight loss. Physical examination indicated tenderness in the epigastrium. Her initial blood tests showed hemoglobin level, 13.4 g/dL; white blood cell count, 5600/μL; platelet count, 281000/μL; and levels of amylase, 48 U/L; lipase, 61 U/L; CA 19-9, 605 U/mL; and CEA, 1.2 ng/mL.

Abdominal computed tomography (CT) and magnetic resonance imaging revealed a 3.5 cm, pancreatic mass abutting the celiac axis, splenic vein, and superior mesenteric artery. There was also hypermetabolic left inguinal lymphadenopathy. She was diagnosed with stage T4N1M0.

**Fig. 1:**
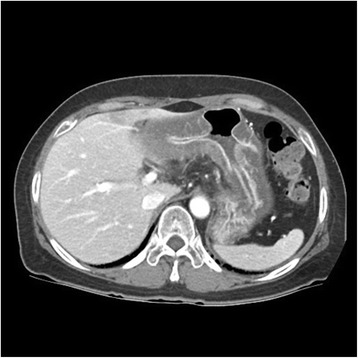
CT shows diffuse low density wall thickening of stomach

**Fig. 2:**
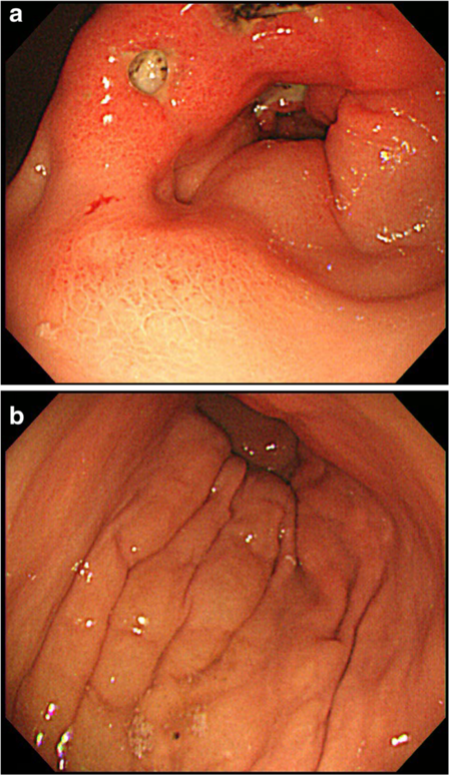
EGD. **a** Several medium sized active ulcers on diffuse, edematous and thickened wall of gastric antrum. **b** Diffuse edematous mucosal swelling on gastric body

**Fig. 3:**
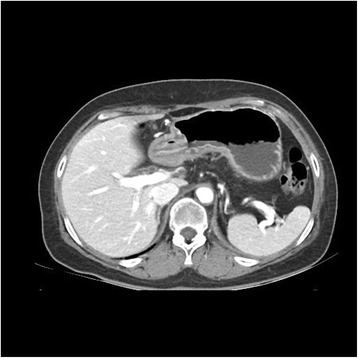
Followed CT scan shows improvement of wall thickening of stomach

**Fig. 4:**
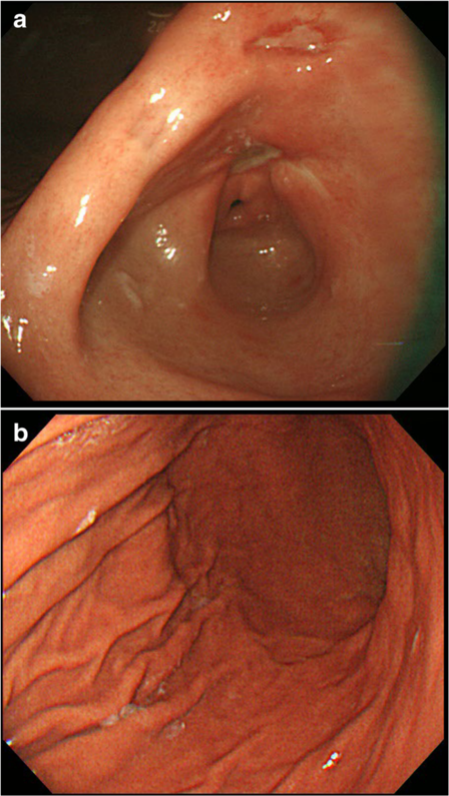
**a**, **b** Followed EGD shows improvement of ulcers and wall thickening

The patient was initially treated with concurrent radiotherapy (total of 50.0 Gy) and chemotherapy of gemcitabine (600 mg/m^2^ weekly for 5 weeks). The patient was then treated with gemcitabine and erlotinib. Gemcitabine was administered at a dose of 1000 mg/m^2^/week for 3 weeks every 4 weeks, and erlotinib (dosed 100 mg once daily) was administered without cessation. After 2 cycles of chemotherapy, the patient visited the emergency room (ER) for epigastric pain, nausea, and vomiting. Abdominal CT scan showed a substantial decrease in the size of the pancreatic mass from 3.5 cm to 2.0 cm, but also revealed diffuse thickening of stomach wall (Fig. 1). The patient underwent an esophagogastroduodenoscopy (EGD). Initial endoscopic findings revealed multiple, deep active ulcers in the gastric antrum (Fig. 2a-b), and microscopic examination of gastric biopsy specimens showed chronic gastritis. The patient discharged on anti-ulcer medication including a PPI. Two months later, she revisited ER with the same symptoms and gastric ulcers still were observed on subsequent EGD. The patient’s symptom had relieved with symptomatic care. Therefore, she received 4 additional cycles of maintenance chemotherapy with gemcitabine and erlotinib and did not have any other side effects with chemotherapy. On the 6-month follow-up examinations, abdominal CT revealed improved thickening of the gastric wall and EGD showed improvement of the gastric ulcers (Figs. 3; 4a-b).

## Discussion

Radiation recall phenomenon is now a well-recognized phenomenon since radiation recall dermatitis was reported in 1975 [4]. Radiation recall dermatitis has been reported most frequently with a frequency of 8.8 % and characterized by an inflammatory reaction within a previously irradiated skin after administration of antineoplatic drugs [5].

Antineoplastic drugs have mainly been involved in radiation recall reactions. Most common anticancer agents that cause radiation recall reactions are the anthracycline doxorubicin, the taxanes docetaxel and paclitaxel, and the antimetabolites gemcitabine and capecitabine [1].

Although there are several pathophysiological theories concerning radiation recall, the most accepted hypothesis is the drug hypersensitivity reaction. In this hypothesis, radiation lowers the inflammatory response threshold and induces the expression of certain cytokines, and then the drug triggers a non-immune inflammatory reaction [1].

Gemcitabine induced radiation recall was reported in the skin, central nervous system, musculoskeletal systems and gastrointestinal tract. The time between initiation of radiation and recall of the radiation phenomenon ranged from 3 weeks to 8 months from the initiation of gemcitabine [6].

Dermatitis and pneumonitis were reported as the radiation recall reactions associated with erlotinib [7]. Recently there was a report of gastrointestinal bleeding secondary to radiation recall gastritis related to erlotinib [3]. The mechanism of erlotinib-induced radiation recall gastritis is also unclear, but Graziani et al. [3] suggested the association between the pathogenesis of radiation recall and erlotinib’s up-regulation of the angiogenic growth factor thymidine phosphorylase. Erlotinib, as an orally administered epidermal growth factor receptor (EGFR) tyrosine kinase inhibitor, can cause gastrointestinal ulcer without relation with radiation because EGFR is highly expressed in the epithelium of the gastrointestinal tract. However, this case showed ulcers and prominent wall thickening only in gastric antrum and body, confined to previously irradiated area. To our knowledge, this report is the first case of radiation recall gastritis due to combination of gemcitabine and erlotinib. It could be assumed that the combination therapy could result in a higher radiation recall incidence.

Radiation recall reactions have been reported in the literature, but management remains controversial whether or not to continue the drug, especially chemotherapeutic agent. Suggested management of the recall reaction is discontinuing drug and initiating steroid therapy, supportive therapy, and/or nonsteroidal anti-inflammatory agents [6]. There were two reports treated with discontinuing drug in radiation gastritis associated with gemcitabine and erlotinib [2, 3]. Meanwhile there is a partial support for the continuation of chemotherapy even when a recall reaction is encountered [8]. In this case, diffuse gastritis and ulcers were treated with PPI and the patient eventually recovered from the radiation recall gastritis without discontinuation of chemotherapy. It can be assumed that recovery was partly due to reduced absorption of erlotinib because absorption of erlotinib is known to be pH dependent. However Ter Heine et al. [9] reported that trough concentrations of erlotinib were not diminished when PPI was administered orally. More studies are needed on the role of PPI in radiation gastritis.

## Conclusions

This is the first case report of radiation recall gastritis due to combination of gemcitabine and erlotinib. It could be followed with proton pump inhibitor during chemotherapy without withdrawal.

## Abbreviations

CA, carbohydrate antigen; CEA, carcinoembryonic antigen; CT, computed tomography; EGD, esophagogastroduodenoscopy; EGFR, epidermal growth factor receptor; ER, emergency room; PPI, proton pump inhibitor
